# Electrochemical determination of closantel in the commercial formulation by square-wave adsorptive stripping voltammetry

**DOI:** 10.1007/s00706-016-1862-z

**Published:** 2016-10-28

**Authors:** Mariola Brycht, Agnieszka Nosal-Wiercińska, Karolina Sipa, Konrad Rudnicki, Sławomira Skrzypek

**Affiliations:** 1 Department of Inorganic and Analytical Chemistry Faculty of Chemistry, University of Lodz, Tamka 12, 91-403 Lodz, Poland; 2 Department of Analytical Chemistry and Instrumental Analysis Faculty of Chemistry, Maria Skłodowska-Curie University, M. Skłodowska-Curie sq. 3, 20-031 Lublin, Poland

**Keywords:** Adsorption, Drug research, Electrochemistry, Reductions, Renewable silver amalgam film electrode, Voltammetry

## Abstract

**Abstract:**

In this paper, the square-wave adsorptive stripping voltammetric (SWAdSV) determination of the veterinary drug closantel using a renewable silver amalgam film electrode (Hg(Ag)FE) is presented. As observed in SWAdSV, closantel provided one well-shaped reduction peak suitable for analytical purposes at potential ca. −1.4 V in the Britton–Robinson (B-R) buffer at pH 7.0. At optimal conditions, the SWAdSV response of Hg(Ag)FE for determining closantel was linear over two concentration ranges of 5.0 × 10^−8^ to 2.0 × 10^−7^ mol dm^−3^ and 2.0 × 10^−7^ to 1.2 × 10^−6^ mol dm^−3^ with a detection limit of 1.1 × 10^−8^ mol dm^−3^. In addition, a relevance of the developed SWAdSV method was successfully verified by the quantitative analysis of closantel in the commercial formulation Closamectin Pour-On with satisfactory results (RSD = 5.8%, recovery = 101.8%). The results showed that the developed procedure can be adequate for screening purposes. Also, the electrochemical behavior of closantel was characterized by cyclic voltammetry, and it was found that closantel exhibited a quasi-reversible behavior with cathodic peak on the forward scan at ca. −1.4 V and anodic peak on the reverse scan at ca. −1.35 V vs. Ag/AgCl in B–R buffer, pH 7.0. As the obtained results showed that the electrode mechanism of closantel is controlled by the adsorption, the effect of adsorption was studied using the electrochemical impedance spectroscopy technique.

**Graphical abstract:**

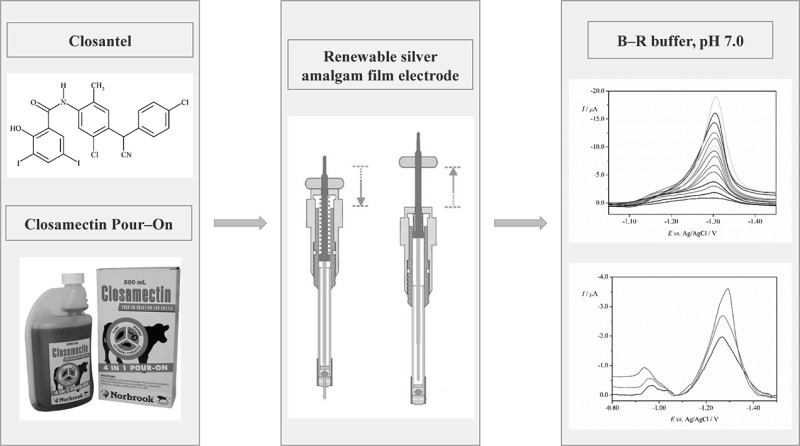

## Introduction

In the last few years, drug prescription has increased several folds, and consequently, an intensification of drug taking by humans and animals has also been observed. It is obvious that medicines play a significant role in the treatment and prevention of diseases in both humans and animals; nevertheless, pharmaceuticals can also cause harmful effects on health and environment [1]. Thus, it has been suggested that the drugs (antibiotics, antiparasitics, antifungals, and anticancer medicines) might prove to be one of the most important environmental pollutions, because these are designed to kill organisms or cells. These pharmaceuticals find their way into rivers, lakes, and even drinking water, and they can have devastating effects on the environment. In most cases, however, the concentrations of drugs found in the environment are much lower than the therapeutic dose; nevertheless, the monitoring of pharmaceuticals in the environment is a very important task [2].

**Fig. 1:**
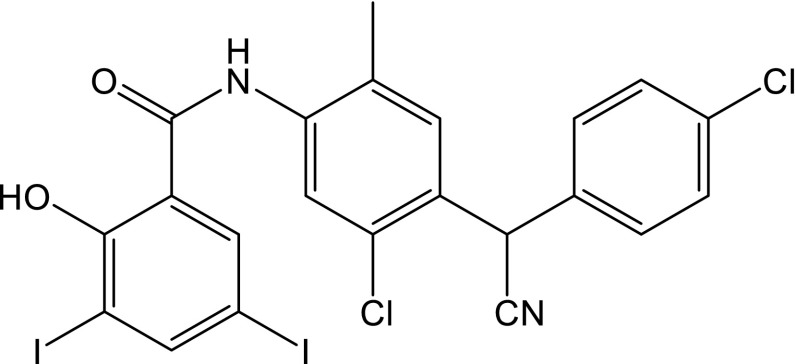
Structure of CLS

Closantel (CLS, *N*-[5-chloro-4-[(4-chlorophenyl)cyanomethyl]-2-methylphenyl]-2-hydroxy-3,5-diiodobenzamide, CAS No. 57808-65-8) is a halogenated salicylanilide derivative with the structure shown in Fig. 1. CLS is classified as a broad-spectrum synthetic antiparasitic drug, which is active against several adult and developmental stages of trematodes, nematodes and arthropods. CLS is mainly used in the prevention and treatment of liver fluke disease in cattle and sheep [3]. CLS decouples the mitochondrial oxidative phosphorylation, which leads to the inhibition of ATP synthesis. That causes a significant change in the energy metabolism, and, as a consequence, the death of the parasite [4]. Although CLS is used as anthelmintic for ruminants, it is contraindicated for humans. It was found that CLS is toxic for humans causing blindness, hematologic and hepatic disorders. Public awareness should be raised about the risks of use of drugs unapproved for human use [4]. This implied the development of suitable analytical methods to determine residues of CLS. Based on the available literature data, there are several studies on the analytical determinations of CLS in pharmaceuticals, biological fluids, agricultural products, and real samples [3, 5–16]. So far, the most frequently applied technique for the CLS determination in the different types of samples has been chromatography, i.e., high-performance liquid chromatography with UV detection (HPLC–UV) [5, 6], HPLC–electrospray ionization tandem mass spectrometry (HPLC–MS/MS) [7], HPLC with fluorescence detection (HPLC–FL) [3, 5, 8–10], ultra high-performance liquid chromatography–tandem mass spectrometry (UPLC–MS/MS) [11], and liquid chromatography–tandem mass spectrometry (LC–MS/MS) [12–16]. Although these methods are obviously sensitive and selective, they are usually time-consuming and relatively expensive. As a possible alternative to the aforementioned analytical methods employed to determine CLS, simple, inexpensive, and rapid voltammetric techniques can also be applied. To the best of our knowledge, no study concerning the electrochemical behavior of CLS has been previously reported in the literature. Thus, this paper is the first report on electrochemical study of CLS.

To date, carbonyl compounds (aldehydes, ketones, quinones) and their nitrogen derivatives were broadly electrochemically investigated, and thus, hundreds of contributions were published during the last few decades [17]. It was found that the carbonyl group belongs to the electron-withdrawing group due to its “lack of electrons”; therefore, the reduction is its typical electrochemical process [17]. It was found that the electrochemical behavior of carbonyl compounds depends strongly on the rest of the molecule and on experimental conditions (protic/aprotic medium, pH, electrode material, presence of other reactant, etc.) [17]. Based on the literature, when only carbonyl group is reduced, the reduction pattern is, in principle, similar and simple, while the mechanism reactions in the presence of other electrochemically active functional groups tend to be much more complicated [17].

In the last two decades, most of the voltammetric methods published in the scientific literature were based on the mercury electrodes. It is obvious that mercury is the best electrode material in analytical practice. Nevertheless, nowadays, there is a tendency to substitute liquid mercury with other less- or non-toxic electrode material, i.e., bismuth-based [18, 19] or amalgam-based electrodes [20–26]. The problem of reducing the amount of mercury used in analytical procedures can be solved with the help of a renewable silver amalgam film electrode (Hg(Ag)FE) [23, 24]. Hg(Ag)FE as a solid amalgam-based electrode is a promising alternative to the family of mercury electrodes due to its features, such as a wide potential window, low noise, and easily mechanically renewable surface. It is worth noting that liquid silver amalgam (a volume not exceeding 10 mm^3^) enables the electrochemical stablility of the electrode for a great number of regeneration cycles, and moreover, Hg(Ag)FE remains exploitable for an extended period of time of several months [23]. To date, Hg(Ag)FE has been widely used in analysis of various organic compounds [27–33].

This paper demonstrates the utility of a renewable silver amalgam film electrode for a rapid, simple, and sensitive determination of CLS. Thus, for the first time, the procedure for the square-wave adsorptive stripping voltammetric determination of CLS based on its reduction signal at Hg(Ag)FE in the commercial formulation Closamectin Pour-On is reported. A note is given to the robustness of the SWAdSV method on the quantitative determination of CLS and to elucidate the nature of CLS at Hg(Ag)FE using a cyclic voltammetric technique. A considerable notice is given to a study of adsorption performed to provide information on the electrochemical processes occurring on the electrode surface by electrochemical impedance spectroscopy technique.

## Results and discussion

### Cyclic voltammetry of CLS on Hg(Ag)FE

**Fig. 2:**
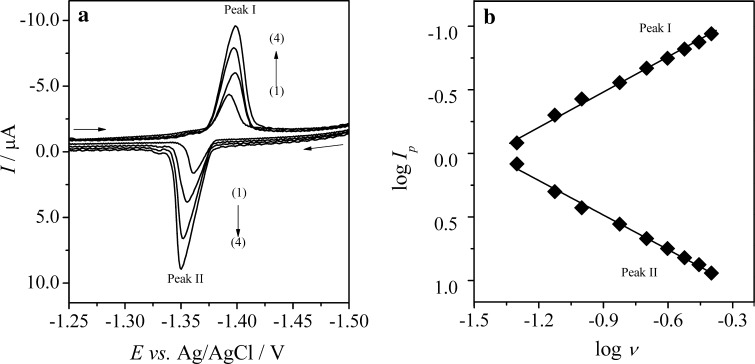
**a** Selected cyclic voltammograms of 1.0 × 10^−5^ mol dm^−3^ CLS recorded on Hg(Ag)FE in B–R buffer, pH 7.0; scan rate (*ν*): (1) 0.1, (2) 0.2, (3) 0.3, and (4) 0.4 V s^−1^; **b** The dependences of the logarithm of the cathodic (*peak I*) and anodic (*peak II*) current (log *I*_*p*_) of CLS on the logarithm of the potential scan rate (log *ν*)

Cyclic voltammetry is the most extensively used technique for obtaining qualitative information about electrochemical reactions. Thus, to acquire a basic knowledge on the electrochemical characteristic of CLS at Hg(Ag)FE and to explain the nature of the electrode process, a detailed CV study was carried out. The cyclic voltammetric behavior of CLS was investigated in the potential window from 0 to −1.6 V at a concentration level of 1.0 × 10^−5^ mol dm^−3^ CLS in B–R buffer, pH 7.0. The potential scan rate (*ν*) was changed from 0.05 to 0.4 V s^−1^ during the experiments. As can be seen from Fig. 2a, two peaks were observed on the CVs; one cathodic peak (peak I) on the forward run (*E*_*p.c*_ = ca. −1.4 V) and one anodic peak (peak II) during the reverse scan (*E*_*p,a*_ = ca. −1.35 V). Also, the peaks currents ratio (*I*_*p,a*_/*I*_*p,c*_) for each scan rate was ca. 1 (vary in the range of 0.9–1.1). Furthermore, *E*_*p,c*_ shifted towards more negative potentials between −1.387 and −1.402 V, while *E*_*p,a*_ moved to more positive potentials between −1.360 and −1.347 V. Therefore, it can be suggested that in this case, an electrochemically quasi-reversible redox couples were observed.

In addition, the cyclic voltammetric technique has been applied to elucidate the mechanisms of the CLS electrode reaction on Hg(Ag)FE. It was found that both anodic and cathodic peak currents were proportional to the scan rates (*ν*), and this is characteristic for an adsorbed substance. Moreover, the plots of log *I*_*p,a*_ and log *I*_*p,c*_ against log *ν* (Fig. 2b) were found to be linear with a slope of 0.91 for cathodic peak and 0.96 for anodic peak, which are close to the expected theoretical slope of 1 for an ideal reaction of surface species. This behavior clearly proves that both cathodic and anodic peak currents were controlled mainly by the adsorption of the electroactive species at the electrode surface.

Since organic molecules containing the carbonyl group (>C=O) in the free form are electrochemically reducible [17, 34, 35], it can be expected that the reduction of CLS, which includes a >C=O group in its chemical structure (Fig. 1), may also result in a similar voltammetric pattern. In addition, it should be noticed that the electrochemically reducible nitrile functional group (–CN) is also present in the structure of CLS; therefore, it can be expected that electrochemical reduction of –CN group can also take place. Nevertheless, it was found that the electroreduction of the carbonyl groups is much easier than nitrile group reduction [17]. Therefore, considering the results of the electrochemical reduction mechanism of the similar compounds [17], it can also be suggested that the voltammetric behavior of CLS in B–R buffer at pH 7.0 recorded at Hg(Ag)FE is presumably caused by a quasi-reversible reduction of the carbonyl group to the hydroxyl group. It can be proposed that the transfer of an electron from the electrode to the carbonyl group is presumably the first electrochemical step, and this is followed by the protonation of radical anion. Further, the reduction of the protonated radical anion generates the –OH group [17]. However, it should be emphasized that a deeper elucidation of the mechanism is beyond the scope of this study.

### Adsorption study

To provide an additional information on the electrochemical processes of CLS, the adsorption study effect on the controlled-growth mercury drop electrode (CGMDE) was performed by AC impedance technique. Due to the excellent reproducibility and polarizability of the CGMDE, this electrode can be successfully used in the adsorption measurements [30, 31, 33, 36]. It can be suggested that the adsorption process at Hg(Ag)FE should occur in the similar way as at the CGMDE. It is connected with the fact that the Hg(Ag)FE is made up of a silver amalgam (liquid mercury layer) with a small amount of silver (1% w/w silver) [23]; thus, it retains the property of mercury electrodes.

**Fig. 3:**
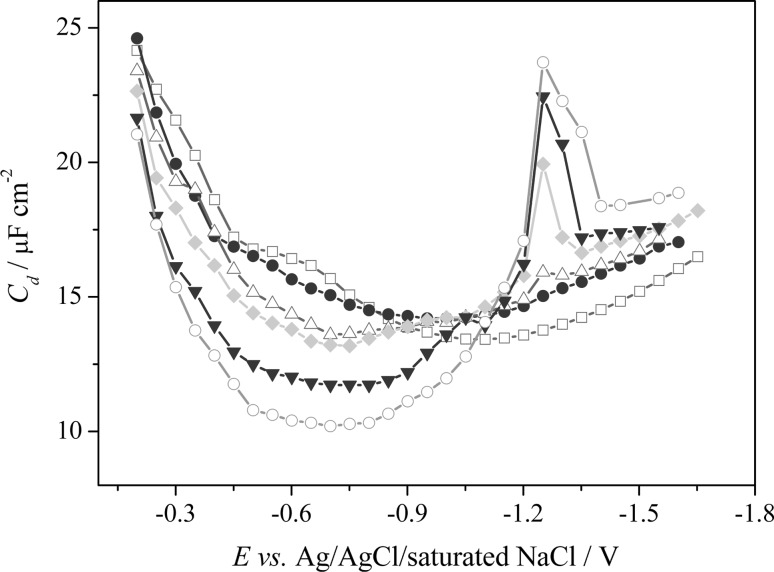
The differential capacity–potential curves of the double-layer interface Hg/B–R buffer (pH 7.0) in the presence of different CLS concentrations; (*unfilled square*) 0, (*filled circle*) 6.0 × 10^−6^, (*unfilled triangle*) 1.0 × 10^−5^, (*filled diamond*) 1.5 × 10^−5^, (*filled inverted triangle*) 3.0 × 10^−5^, (*unfilled circle*) 5.0 × 10^−5^ mol dm^−3^

The results showed that on the differential capacity curve obtained for the supporting electrolyte (B–R buffer, pH 7.0) without CLS (Fig. 3, unfilled square), outline maximum occurred (*E*_*p*_ ≈ −0.6 V), and it can be related to the change of the orientation of the water molecules in the double layer [31, 37, 38]. The presence of CLS in the supporting electrolyte solution caused a significant decrease of the differential capacity in the studied potential range (from −0.2 to −1.0 V). It should be emphasized that the adsorption peaks of CLS did not occur on the differential capacity curves in the investigated potential range, while at the *E*_*p*_ = −1.35 V, and in the CLS concentrations higher than 1.0 × 10^−5^ mol dm^−3^, the desorption peak seems to be observed. Moreover, the height of the desorption peak increases with the increasing concentration of adsorbate in the supporting electrolyte solution. Such changes of the double-layer differential capacity are related to strong adsorption of CLS molecules on the mercury surface [30, 31, 37, 38].

### Optimization of experimental conditions

Due to the fact that a method is viewed as incomplete and cannot be considered as reliable in routine use if no validation test was performed [35, 36], a robustness test in the validation procedure should be studied as part of the procedure development. Therefore, to establish the optimal conditions for voltammetric determination of CLS on Hg(Ag)FE, and to gain the knowledge about its redox behavior, thoroughgoing studies were carried out.

#### The influence of pH of the supporting electrolyte

**Fig. 4:**
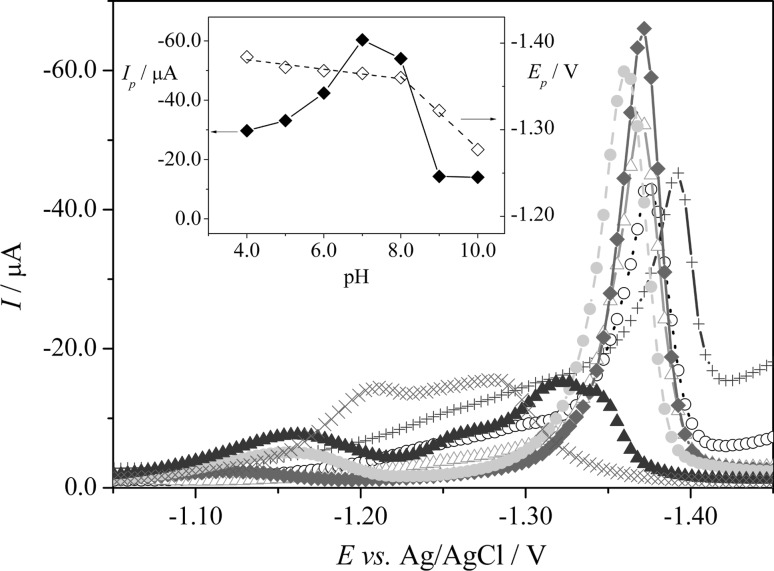
Selected SW voltammograms of CLS (5.0 × 10^−6^ mol dm^−3^) on Hg(Ag)FE in B–R buffers, pH 2.0–12.0; (+) 4.0, (*unfilled circle*) 5.0, (*unfilled triangle*) 6.0, (*filled diamond*) 7.0, (*filled circle*) 8.0, (*filled triangle*) 9.0, and (×) 10.0; (B) The *inset* shows the dependence of the SWV peak currents (*I*_*p*_) (*filled diamond*, left *y* axis) and peak potentials (*E*_p_) (*unfilled diamond*, right *y* axis) on pH

In the present work, the effect of the Britton–Robinson (B-R) buffer over the pH range from 2.0 to 12.0 was investigated for its suitability in the determination of CLS. As it can be seen from Fig. 4, CLS provided one reduction peak at negative potential at ca. −1.4 V in pH range from 4.0 to 8.0, whereas in pHs higher than 8.0, signal is not made by a simple single reduction, but seems to consist of two overlapping peaks (the evaluation of the peak current was done by a tangent fit and enumeration of the highest current in the tangent range). At pH values ≤3.0 and ≥10.0, no voltammetric peaks of CLS were recorded (results not shown). As it can be seen from the inset of Fig. 4 (filled diamond, left *y* axis), a peak intensities enhancement was observed within the pH range from 4.0 to 7.0. The maximum was achieved at pH 7.0, and then, peak intensities decrease in the pH range from 7.0 to 10.0. The best results were achieved at pH 7.0 for B–R buffer. Also, the effect of the buffer dilution from 40 to 100% volume of buffer in the voltammetric cell on the peak current of CLS was checked. In our case, the results showed that the pH was almost stable and varied slightly from 6.91 (*V*_*buf*_:*V*_*H2O*_ = 4:6) to 6.96 (100% of buffer). The samples containing 50% of B–R buffer at pH 7.0 and 50% of water were used in all further voltammetric experiments.

It should also be noticed that the increase in pH caused a shift in the position of the reduction peak towards less negative values from −1.38 to −1.28 V in the pH range of 4.0–10.0 (the inset of Fig. 4, unfilled diamond, right *y* axis). The evolution of the peak potential (*E*_*p*_) with pH showed two linear segments. In the pH range of 4.0–8.0, the peak potential indicated a very slight linear variation (practically negligible) with pH (from −1.38 to −1.36 V) with a slope of −5.5 mV pH^−1^, according to Eq. (1):

1 $$E_{p}/V \, = 0.0055{\text{ pH }} - \, 1.4028 \quad {\text{R}}^{2} = \, 0.9920$$

In the pH range of 8.0–10.0, the peak potential shifted linearly towards less negative value (from −1.36 to −1.28 V) with the slope corresponding to 4.2 mV pH^−1^, according to Eq. (2):

2 $$E_{p}/V \, = 0.042{\text{ pH }} - \, 1.693 \quad {\text{R}}^{2} = \, 0.9976$$

#### Optimization of the SWV parameters

Further, the optimization of the experimental parameters for the voltammetric determination of CLS with SWAdSV at Hg(Ag)FE was performed to identify the conditions at which the observed maximum peak height was accompanied by the best signal shape and height. The optimization was carried out by varying the conditioning potential (*E*_*cond*_) and time (*t*_*cond*_), the SW amplitude (E_*SW*_), the SW frequency (*f*), and the step potential of the staircase waveform (Δ*E*). During adjustment of the aforementioned parameters, each of them was changed, while the others were kept fixed using 5.0 × 10^−6^ mol dm^−3^ CLS concentration.

According to current understanding [28, 30–32], the conditioning potential and time can significantly influence the voltammetric response of analyzed compound at Hg(Ag)FE in aqueous solutions. It should be noted that conditioning denotes electrolytic cleaning of the electrode surface, and the conditioning step is performed to remove contaminants or materials from the electrode surface. Therefore, at first, the effect of the conditioning potential on the voltammetric signals of CLS (5.0 × 10^−6^ mol dm^−3^) was investigated at *t*_*cond*_ of 15 s in the range from −1.4 to −2.0 V. The best results were obtained at the conditioning potential of −1.50 V; thus, this value was chosen for subsequent investigations. Further, the impact of *t*_*cond*_ on the behavior of CLS (5.0 × 10^−6^ mol dm^−3^) was also tested. The conditioning time parameter was varied in the range of 0–50 s. The optimum results were obtained for conditioning time of 10 s; therefore, further analytical studies were carried out at *t*_*cond*_ of 10 s.

Next, the influence of the height of SW pulses was the first critical choice. *E*_*SW*_ was investigated in the range from 10 to 140 mV. In the entire investigated range, the current of the peak increased linearly with increasing *E*_*SW*_; nevertheless, amplitudes higher than 40 mV caused a distorted peak shape. It should be noted that larger amplitude values yield a larger response, but faradaic peaks become broader and potential resolution was lost at very large amplitudes. Therefore, *E*_*SW*_ = 40 mV was applied in the subsequent study. Further, the impact of the SW frequency (*f*) on the peak current was also tested in the range of 10–150 Hz. It should also be mentioned that the use of a higher *f* would have given a higher response; nevertheless, the growing capacitive current and an uncompensated ohmic drop effect would be observed. Due to the aforementioned facts, the frequency of 110 Hz was used as the optimum for further analytical studies. The last parameter of potential modulation, the step potential of the staircase waveform (Δ*E*_*s*_) was studied in the range of 1–10 mV. The results showed that by increasing the Δ*E*_*s*_, the peak current remained almost unchanged; nevertheless, a higher step potential causes a distortion of peak shape. The best-formed signal was obtained for a step potential of 1 mV, and this value was applied in subsequent studies.

After the conditioning stage, the stirring of the solution was stopped, and the solution was allowed to reach equilibrium. Therefore, the investigation of the effect of *t*_*eq*_ was also performed. The equilibration time values were changed in the range from 5 to 40 s, and the obtained results shown that the equilibration time influenced the peak current. The best results were obtained for *t*_eq_ = 15 s.

#### Influence of the accumulation parameters

In addition, the analytical sensitivity can be enhanced by combining the SWV technique with adsorptive deposition. Thus, to improve the sensitivity and detection limit of the method, the influence of the accumulation potential (*E*_*acc*_) and accumulation time (*t*_*acc*_) was investigated. The effect of the deposition potential on the stripping peak current (2.5 × 10^−6^ mol dm^−3^) was examined over the range from 0 to −1.2 V with a fixed accumulation time of 10 s. It was found that when *E*_*acc*_ was between 0 V and −1.0 V, the peak intensity was generally unchanged, whereas at potentials lower than −1.0 V, the reduction signal intensity was decreased. To select the optimum accumulation potential, the dependence of the accumulation time on the peak current was checked in the range from 0 to 100 s at a CLS concentration level of 2.5 × 10^−6^ mol dm^−3^ for the *E*_*acc*_ of −0.5, −0.7, and −1.0 V. It was found that the observed response was highly sensitive to the deposition time. The best results were registered for CLS at *E*_*acc*_ = −1.0 V. For the selected accumulation potential of −1.0 V, the current response increased with the accumulation time from 0 to 40 s, reaching almost a steady state when CLS was preconcentrated for a duration between 40 and 100 s. This behavior suggested a saturation of accessible adsorption of CLS on the Hg(Ag)FE. The optimum accumulation time to the electrode surface saturation was 30 s.

### Analytical characteristic

A voltammetric procedure for determination of CLS using the reduction peak at a potential of ca. −1.4 V at Hg(Ag)FE by employing SWAdSV was performed under optimized conditions. The proposed procedure for the determination of CLS was validated. It should be noticed that the validation of an analytical method is the process by which it is established, by laboratory studies, that the performance characteristics of the method meet the requirements for the intended analytical applications. Therefore, the method validation parameters, such as linearity and range, sensitivity, limit of detection (LOD), limit of quantification (LOQ), precision (repeatability), and accuracy (recovery), were estimated.

**Fig. 5:**
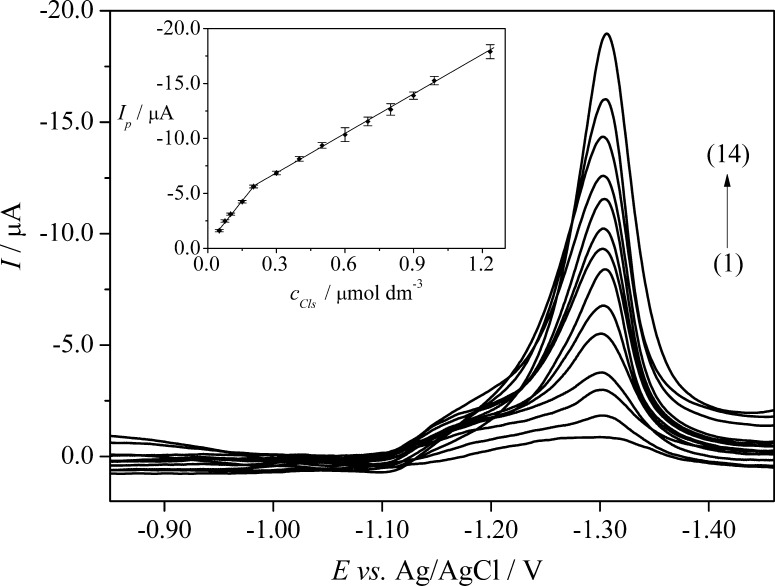
SWAdS voltammograms of CLS in B–R buffer, pH 7.0. The *inset* shows the corresponding calibration graphs; LDR_1_ (1–5): (1) 5.0 × 10^−8^, (2) 7.5 × 10^−8^, (3) 1.0 × 10^−7^, (4) 1.5 × 10^−7^, (5) 2.0 × 10^−7^ mol dm^−3^; LDR_2_ (5–14): (5) 2.0 × 10^−7^, (6) 3.0 × 10^−7^, (7) 4.0 × 10^−7^, (8) 5.0 × 10^−7^, (9) 6.0 × 10^−7^, (10) 7.0 × 10^−7^, (11) 8.0 × 10^−7^, (12) 9.0 × 10^−7^, (13) 9.9 × 10^−6^, and (14) 1.2 × 10^−6^ mol dm^−3^. The error bars were constructed as confidence intervals (*t*_(*p*=95%, *n*=4)_ = 3.18)

The reliability of the SWAdSV technique for the quantification of CLS was tested by calibration curve constructed by plotting reduction peak current (*I*_*p*_) against the increasing concentrations of CLS (*c*_*CLS*_) in quadruplicate (*n* = 4), and the corresponding SWAdSVs are depicted in Fig. 5. As it can be seen from Fig. 5, the reduction peak current increases linearly in two dynamic linear ranges (*LDR*) of 5.0 × 10^−8^ to 2.0 × 10^−7^ mol dm^−3^ (*LDR*_1_) and 2.0 × 10^−7^ to 1.2 × 10^−6^ mol dm^−3^ (*LDR*_2_). The aforementioned linear relationships can be expressed by Eqs. (3) and (4):

3 $$LDR_{1} I_{p} /\mu A \, = \, - 26.13c/\mu {\text{moldm}}^{ - 3} - \, 0.42 \quad R^{2} = \, 0.9975$$

4 $$LDR_{2} I_{p} /\mu A \, = \, - 11.89c/\mu {\text{moldm}}^{ - 3} - \, 3.29 \quad R^{2} = \, 0.9992$$

The appearance of the two linear ranges suggests an adsorption process. For lower concentrations, the adsorption process did not alter the kinetics of the electrode surface, whereas, for higher concentrations, the peak current intensity decreased due to saturation of the electrode surface by the electroactive species. The proof of the aforementioned conclusions is the desorption peak observed at a concentration of 1.0 × 10^−5^ mol dm^−3^ on the differential capacity curves (Fig. 3), which was increased with further increase of the concentration of CLS in the supporting electrolyte solution. Moreover, it should be noticed that the peak potentials recorded in lower CLS concentrations were shifted towards less negative potential (−1.3 V, Fig. 5) compared to higher CLS concentrations (*E*_*p*_ about −1.4 V, Fig. 4).

The limits of detection (LOD) and quantification (LOQ) were calculated from the calibration curve (*LDR*_1_) as described in Experimental section. The parameters of the calibration straight lines for the quantitative determinations of CLS obtained using SWAdSV at Hg(Ag)FE are summarized in Table 1. The low detection limit was obtained as a consequence of the high signal-to-noise ratio.

**Table 1:** Parameters of the calibration straight lines for the quantitative determinations of CLS by SWAdSV at Hg(Ag)FE in B–R buffer, pH 7.0

Linear dynamic range/µmol dm^−3^	LDR_1_	LDR_2_
	0.05–0.2	0.2–1.2
Coefficient of determination (*R* ^*2*^)	0.9975	0.9992
Slope (*b*)/µA dm^3^ µmol^**−**1^	−26.13	−11.89
Standard deviation of slope (SD_b_)/µA dm^3^ µmol^**−**1^	0.58	0.31
Intercept (*a*)/µA	−0.42	−3.29
Standard deviation of intercept (SD_a_)/µA	0.024	0.095
Limit of detection (LOD)/µmol dm^−3^	0.011	
Limit of quantification (LOQ)/µmol dm^−3^	0.037	

The precision (repeatability) of the analytical signal was tested by recording SWAdSV curves for each studied concentration of CLS (*n* = 4) in both linear dynamic ranges. To check the correctness of the method, the precision and accuracy of the SWAdSV method were also calculated for the different concentrations of CLS in the linear dynamic ranges. The results are summarized in Table 2, and they confirm high precision of the proposed SWAdSV method. It is obvious that the developed method is suitable for the CLS determination.

**Table 2:** Repeatability of CLS peak current, precision and recovery at various concentrations of CLS obtained by SWAdSV on Hg(Ag)FE in B–R buffer, pH 7.0

Added/µmol dm^−3^	RSD of *I*_*p*_/%	Found/µmol dm^−3a^	RSD/%	Recovery/%^b^
LDR_1_				
0.05	4.3	0.046 ± 0.004	5.9	92.3
0.075	2.7	0.079 ± 0.004	3.3	105.0
0.1	2.0	0.102 ± 0.004	2.3	102.0
0.15	1.6	0.148 ± 0.004	1.8	98.7
0.2	1.5	0.200 ± 0.005	1.6	100.0
LDR_2_				
0.2	1.5	0.20 ± 0.01	3.5	99.4
0.3	1.3	0.30 ± 0.01	2.9	99.1
0.4	1.6	0.41 ± 0.02	2.7	101.5
0.5	1.7	0.51 ± 0.02	2.6	102.9
0.6	3.8	0.59 ± 0.05	5.6	98.7
0.7	2.2	0.69 ± 0.03	3.0	99.0
0.8	2.6	0.79 ± 0.04	3.5	98.8
0.9	1.5	0.89 ± 0.03	1.9	99.0
1.0	1.6	1.01 ± 0.03	2.0	101.6
1.2	2.2	1.23 ± 0.05	2.7	100.0

^a^Mean value ± confidence interval, *t*_(*p*=95%, *n*=4)_ = 3.18

^b^Recovery = 100% + [(Found − Added)/Added] × 100%

### Application to analysis of the commercial formulation Closamectin Pour-On

**Fig. 6:**
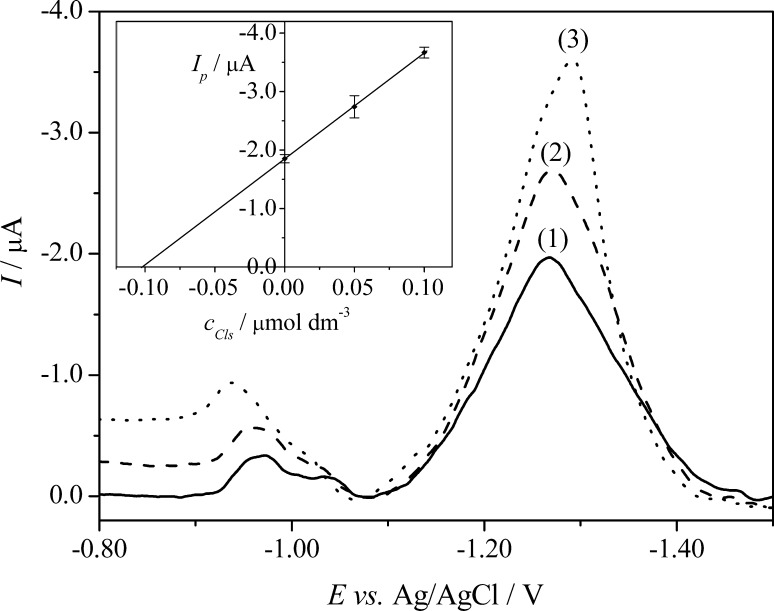
SWAdS voltammograms of CLS determination in the commercial formulation Closamectin Pour-On using the standard addition method; (1) the commercial formulation sample, (2) as (1) + 5.0 × 10^−8^ mol dm^−3^; and (3) as (1) + 1.0 × 10^−7^ mol dm^−3^. The* error bars* were constructed as confidence intervals (*t*_(*p*=95%, *n*=4)_ = 3.18)

Subsequently, to check the correctness of the developed SWAdSV method, the procedure was successfully applied to determine CLS in the commercial formulation Closamectin Pour-On at the concentration level from the linear concentration range. The preparation of samples is mentioned in the Experimental section. It was found that the SWAdSVs of Closamectin Pour-On (Fig. 6, curve (1)) exhibited three reduction peaks at potentials −0.95, −1.05, and −1.25 V. As mentioned in the Experimental section, the commercial formulation Closamectin Pour-On contains 200 mg cm^−3^ of closantel (as closantel sodium dihydrate) as well as 5 mg cm^−3^ ivermectin and 0.1 mg cm^−3^ brilliant blue FCF (E133). Therefore, it can be stated that the highest signal at *E*_*p*_ = −1.25 V was connected with the reduction of CLS (due to the highest concentration of CLS in the formulation), while the peaks at *E*_*p*_ = −0.95 and −1.05 V were probably related to the reduction of ivermectin or/and brilliant blue.

The applicability of the procedure (four replicate determinations, *n* = 4) was tested using the standard addition method. The average values of the obtained results are shown in Table 3, and RSD showed high repeatability of determination. The obtained results indicate that the proposed procedure using Hg(Ag)FE is feasible for the determination of CLS in the commercial formulation. The example of SWAdS voltammograms for the CLS determination in Closamectin Pour-On is shown in Fig. 6.

**Table 3:** Analytical results of the determination of CLS by the SWAdSV procedure (*n* = 4)

Sample	Added/µmol dm^−3^	Found/µmol dm^−3a^	*RSD*/%	Recovery/%
Closamectin Pour-On	0.1	0.102 ± 0.009	5.8	101.8

^a^Mean value ± confidence interval, *t*_(*p*=95%, *n*=4)_ = 3.18

## Conclusion

This paper presents a novel and alternative electroanalytical method for the determination of closantel at Hg(Ag)FE. The electrochemical behavior of CLS was established and studied for the first time. The proposed electroanalytical method, performed under optimized conditions, exhibits the limit of detection in the 10^−8^ mol dm^−3^ concentration range in 0.02 mol dm^−3^ Britton–Robinson buffer, pH 7.0. The obtained results showed that Hg(Ag)FE can be a useful tool for the determination of CLS in the commercial formulation without any sample preparation, except dilution. Moreover, the SWAdSV method elaborated offers the sensitive determination of CLS, providing an adequate repeatability and recovery. Thus, the Hg(Ag)FE can be directly applied for a field analysis due to its mechanical stability and easy regeneration of film in contrast to the classical hanging mercury drop electrode. Moreover, the proposed method can serve as a good alternative to HPLC, GC, LC–MS, and other analytical techniques used for pesticide analysis. In the present work, considerable attention was given to the explanation of the electrode mechanism of CLS at Hg(Ag)FE by cyclic voltammetry, and the obtained results showed that the electrode mechanism of CLS is a quasi-reversible process controlled by the adsorption of closantel. To provide information on the electrochemical processes occurring on the electrode surface, a detailed adsorption study using electrochemical impedance spectroscopy (EIS) technique has been performed, and the results showed that a strong adsorption of CLS molecules occurs on the mercury surface.

## Experimental

### Apparatus

The SWAdSV and CV studies were carried out using a µAutolab type II potentiostat–galvanostat (EcoChemie Autolab B.V., Utrecht, the Netherlands) connected to a computer with GPES software (General Purpose Electrochemical System, version 4.9) for data acquisition, and an M164 electrode stand (MTM Anko Instruments, Cracow, Poland). The experiments were performed in three-electrode electrochemical cell consisting of reference electrode (a silver/silver chloride electrode, Ag/AgCl, 3 mol dm^−3^ KCl, Mineral, Poland) and counter electrode (a platinum wire, Pt, 99.99%, The Mint of Poland, Warsaw, Poland). A renewable silver amalgam film electrode (Hg(Ag)FE, surface area: 0.12 cm^2^, AGH University of Science and Technology, Cracow, Poland) was used as a working electrode [23, 24].

Adsorption measurements were carried out using different set from the one used in SWAdSV analysis. The adsorption studies were measured with an electrochemical analyzer µAutolab type III (Eco Chemie, Utrecht, the Netherlands) controlled by GPES software. The investigations were conducted in a three-electrode cell with a hanging controlled-growth mercury drop electrode (CGMDE, Entech, Cracow, Poland) as the working electrode (electrode area: 0.0162 cm^2^, drop time: 3 s), Ag/AgCl/saturated NaCl (Corning type, Fluka, Poland) as the reference electrode, and a platinum spiral (Pt, 99.99%, UMCS, Lublin, Poland) as the counter electrode.

An Orion Star pH meter (Model A111, Thermo Scientific, the Netherlands) with a pH electrode (type Polilyte Lab, Hamilton, Switzerland) was used to check the pH of the solutions.

### Reagents and solutions

All chemicals used were of analytical reagent grade. A fresh CLS stock solution (1.0 × 10^−4^ mol dm^−3^) was prepared weekly by dissolving an exact weight of the analytical standard of CLS (97.3% purity, PESTANAL^®^, Fluka, Poland) in acetonitrile with the help of an ultrasonic bath (5 min). The prepared stock solution of CLS was stored in a refrigerator prior to analysis, and the changes in stability of this solution were not observed during five days. The Britton–Robinson buffer (B-R) solutions were prepared by mixing the same concentrations (0.04 mol dm^−3^) of orthophosphoric acid, boric acid, and acetic acid in triply distilled water, and adjusting to the desired pH values (in the pH range of 2.0–10.0) with the sodium hydroxide solution (0.2 mol dm^−3^). Argon (5 N, Linde gas, Poland) and nitrogen (99.999%, Air Products, PRM) in voltammetric and adsorption measurements, respectively, were used without further purification. All electrochemical measurements were conducted at the laboratory temperature. The commercial formulation Closamectin Pour-On (Norbrook Laboratories Limited, North Ireland) contains 200 mg cm^−3^ of closantel (as closantel sodium dihydrate) as well as 5 mg cm^−3^ of ivermectin and 0.1 mg cm^−3^ of brilliant blue FCF (E133).

### Pretreatment of Hg(Ag)FE

Chemical activation of electrode surface in 2% HNO_3_ for about 5 min was applied if the loss of the response for the electrode was observed. Moreover, the procedure of mechanical refreshing of the silver amalgam film at the Hg(Ag)FE (the formation of a new layer of the amalgam film) [23, 24], as well as the electrochemical conditioning of the electrode surface was performed before each scan. The application of a specified negative potential to the working electrode for a certain time (the conditioning step) is recommended to ensure the removal of oxygen and oxides adsorbed on the electrode surface during the formation of the amalgam film, to remove the components adsorbed on the surface of the electrode, as well as to decrease the residual current and to subsequently achieve low detection limits of the stripping voltammetric quantification [23].

### Voltammetric procedure

In the present paper, the B–R buffer (pH 7.0) was used as the supporting electrolyte. Optimized square-wave adsorptive stripping voltammetric parameters were as follows: conditioning potential of −1.5 V, and conditioning time of 10 s, SW amplitude of 40 mV, SW frequency of 110 Hz, SW step potential of 1 mV, equilibration time of 15 s, accumulation potential of −1.0 V, and accumulation time of 30 s. In cyclic voltammetry (CV), the effect of the scan rate was tested in the range from 0.05 to 0.4 V s^−1^ in the potential range from 0 to −1.6 V.

To obtain the blank signal, 10 cm^3^ of the supporting electrolyte consisting of 5 cm^3^ of the appropriate B–R buffer and 5 cm^3^ of water were transferred into the voltammetric cell. Next, the solution was purged with pure argon for 600 s to remove oxygen in the cathodic potential window. Argon was passed over the solution during the entire measurements. After mechanical refreshing of the silver amalgam film, a conditioning step (the application of an appropriate negative potential for a certain period of time) was carried out. Further, the deposition step was applied in stirred solution by applying a constant potential for a certain period of time. After the equilibrium time, a negative ongoing potential scan was applied. To record the voltammograms of CLS, the required volumes of CLS were added to the cell, and the solution was again purged with argon for a further 60 s. All voltammograms were recorded under the inert atmosphere of the cell.

The calibration curve was prepared under optimized SWAdSV parameters. The following procedure was used: the successive addition of the stock solution of CLS was made into the cell with 10 cm^3^ of the supporting electrolyte. The paired *t* test at the 95% confidence interval (*t*_(*p*=95%, *n*=4)_ = 3.18) and the method of least squares linear regression were used. The detection (LOD) and quantification (LOQ) limits were calculated from the calibration curve (LDR_1_) based on the following equations: LOD = 3 SD/*b* and LOQ = 10 SD/*b*, respectively. Standard deviations (SD) were calculated for the intercept (four runs) and *b* stands for the slope of the calibration graph [39, 40].

All voltammograms were baseline-corrected using the moving average with a peak width of 2 mV included in GPES 4.9 software. This treatment helps in improving visualization and identification of peaks.

### Adsorption procedure

The electrochemical impedance spectroscopy (EIS) technique was used to measure the double-layer capacity (*C*_*d*_) with the reproducibility of the capacity measurements (the average value) of ±0.5%. The capacity dispersion was studied at different frequencies between 200 and 1000 Hz (for the whole polarization range). To obtain proper equilibrium values of differential capacity, linear dependence of capacity *vs.* square element from frequency was extrapolated to zero frequency.

To conduct the adsorption measurements, the following procedure was used: 10 cm^3^ of the supporting electrolyte was transferred to the electrochemical cell and the solution was purged using nitrogen for 300 s. If some reagents were added, the solution was purged again with nitrogen for 30 s. Nitrogen was passed over the solution during the measurements.

### Preparation of the commercial formulation Closamectin Pour-On samples

The declared CLS concentration in the commercial formulation Closamectin Pour-On was 200 mg cm^−3^, i.e., 0.2774 mol dm^−3^. Due to the high concentration of CLS in the fungicidal preparation, appropriate dilution was required to obtain a CLS concentration suitable for voltammetric linear concentration response. No extraction steps were undertaken prior to the voltammetric analysis. A sample of 36 mm^3^ was transferred to a 100-cm^3^ flask, and further diluted with water to arrive at a concentration of 1.0 × 10^−4^ mol dm^−3^. Next, 5 mm^3^ of a sample was transferred to a voltammetric cell with supporting electrolyte. The final CLS concentration in the cell was 5.0 × 10^−8^ mol dm^−3^. When the SWAdSVs of the fungicidal formulation samples were recorded, consecutive doses of the stock solution of CLS were added to the voltammetric cell (the concentrations were 5.0 × 10^−8^, and 1.0 × 10^−7^ mol dm^−3^). Voltammograms were recorded after each addition. The results were analyzed using the paired *t* test at 95% confidence interval (*t*_(*p*=95%, *n*=4)_ = 3.18).
